# A Pipeline for High-Throughput Concentration Response Modeling of Gene Expression for Toxicogenomics

**DOI:** 10.3389/fgene.2017.00168

**Published:** 2017-11-01

**Authors:** John S. House, Fabian A. Grimm, Dereje D. Jima, Yi-Hui Zhou, Ivan Rusyn, Fred A. Wright

**Affiliations:** ^1^Bioinformatics Research Center, North Carolina State University, Raleigh, NC, United States; ^2^Center for Human Health and the Environment, North Carolina State University, Raleigh, NC, United States; ^3^Department of Veterinary Integrative Biosciences, Texas A&M University, College Station, TX, United States; ^4^Department of Biological Sciences, North Carolina State University, Raleigh, NC, United States; ^5^Department of Statistics, North Carolina State University, Raleigh, NC, United States

**Keywords:** expression-based dose–response modeling, dose–response modeling, bioinformatics-pipeline, toxicogenomics, bioinformatics & computational biology, iPSCs, cardiomyocytes, expression profiling

## Abstract

Cell-based assays are an attractive option to measure gene expression response to exposure, but the cost of whole-transcriptome RNA sequencing has been a barrier to the use of gene expression profiling for *in vitro* toxicity screening. In addition, standard RNA sequencing adds variability due to variable transcript length and amplification. Targeted probe-sequencing technologies such as TempO-Seq, with transcriptomic representation that can vary from hundreds of genes to the entire transcriptome, may reduce some components of variation. Analyses of high-throughput toxicogenomics data require renewed attention to read-calling algorithms and simplified dose–response modeling for datasets with relatively few samples. Using data from induced pluripotent stem cell-derived cardiomyocytes treated with chemicals at varying concentrations, we describe here and make available a pipeline for handling expression data generated by TempO-Seq to align reads, clean and normalize raw count data, identify differentially expressed genes, and calculate transcriptomic concentration–response points of departure. The methods are extensible to other forms of concentration–response gene-expression data, and we discuss the utility of the methods for assessing variation in susceptibility and the diseased cellular state.

## Introduction

Among the key challenges in contemporary toxicity testing is addressing increasing numbers of commodity chemicals with insufficient toxicity characterization, a trend that is at least partially attributable to the limitations associated with *in vivo* testing strategies. Additional challenges are associated with animal to human extrapolation, as well as concerns over the ethics and expense of animal testing. These challenges were described in the National Toxicology Program’s (NTP) 2004 *Vision and Roadmap for the 21st Century*, and the National Research Council’s (NRC) report on *Toxicity Testing in the 21st Century* (National Research Council, 2007), which envisioned a strategic shift from exclusive reliance on animal-derived data in chemical regulation to the implementation of novel data streams, including high-throughput *in vitro* testing, omics data, and computational modeling. More recently the NRC report, *A Framework to Guide Selection* of Chemical Alternatives (National Research Council, 2014) and the National Academies report, *Using 21st Century Science to Improve Risk-Related Evaluations* (National Academies, 2017), articulated the need to transition from expensive and incomplete animal testing to high-throughput exposure assessment of new and existing chemicals. Thus, the use of novel data sources to conduct human and animal health risk assessment, including genomic, epigenomic, cell, and *in silico*-based streams, has become imperative.

Gene-expression data are also important in evaluating effects of chemicals on cells and tissues. The Library of Integrated Network-Based Cellular Signatures (LINCS) is a database of over 1 million gene expression signatures, or perturbations, generated using a targeted hybridized bead-base flow sorter (Peck et al., 2006; Duan et al., 2014) from either drug/chemical exposure or biological knockdown/knockout with 50 different cell types (Campillos et al., 2008) on a ∼1,000 gene-set with full-transcriptome imputation. The related Connectivity Map (Lamb et al., 2006), a database of transcriptional multiplexed microarray technology from multiple cancer cell lines exposed to ∼5,000 drugs and small-molecule compounds, has since been combined into the NIH LINCS database. Further, the DrugMatrix^®^ database contains, in addition to a myriad of phenotypic endpoints, microarray gene-expression data in the rat for over 600 different compounds in multiple tissues. Lastly, the Toxicogenomics Project-Genomics Assisted Toxicity Evaluation Systems (TG-GATEs) database has compiled toxicological endpoints and gene expression data from rats (*in vivo* and *in vitro* primary hepatocytes) and humans (*in vitro* primary hepatocytes) on 170 hepato- and renal-toxicants at multiple doses and time points (Uehara et al., 2010; Igarashi et al., 2015).

The advent of next-generation sequencing (NGS) technology has allowed for dramatic advances in the characterization of genomic, epigenomic, and gene expression endpoints. In contrast to reverse transcriptase PCR and microarrays, NGS results in reduction or elimination of numerous sources of variation (Okoniewski and Miller, 2006; Klebanov et al., 2007; Royce et al., 2007). Anticipating a reduced cost in interrogating only a portion of the transcriptome, phase III of the Tox21 initiative has included the development of the S1500 human gene-set (Merrick et al., 2015) to use for chemical and drug screening. This S1500 gene-set was designed to be representative of the human transcriptome, inclusive of the original L1000 (LINCS) gene-set, and optimized for pathway coverage and co-expression information. The main goal is to represent diversity of expression response to disease and chemical exposure in a cost-effective manner.

RNA-Seq, although considered the gold standard for gene expression (Ellis et al., 2013), does have shortcomings. These include bias introduced during mRNA enrichment and library preparation (Han et al., 2015), as well as substantial monetary, computing hardware, and bioinformatics costs. Targeted sequencing technology, such as TempO-Seq^TM^ (Templated Oligo assay with Sequencing readout), which was originally adapted from RASL-seq (Li et al., 2012), specifically targets unpurified RNA in cellular lysates. Two detector oligos are used that can only be ligated when hybridized next to each other on RNA, and confer specificity and eliminate positional bias introduced by poly-(A)+ selection. Sequencing and sample-specific adapters are then hybridized to the original probes for sequencing (Yeakley et al., 2017), allowing for assessment of differentially expressed transcripts in a high-throughput manner while alleviating some of the shortcomings of untargeted RNA-seq. These potential improvements in cost and reduction in sources of variation are attractive for high-throughput concentration–response transcriptomic profiling.

To date, much of the effort to characterize toxicity transcriptomic endpoints has focused on individual concentration–responses from drugs and a small number of environmental chemicals. Targeted sequencing allows for more economical interrogation of the transcriptome and opens the door for high-quantity, high-throughput assessment of drug/chemical concentration–response. We report here a pipeline for utilizing TempO-Seq (BioSpyder Technologies, Inc., Carlsbad, CA, United States), a targeted RNA sequencing technology, to assess gene-transcript concentration–response relationships to chemical exposure. Much of the pipeline is extensible to any transcriptional profiling of chemical response, where sample sizes are likely to be modest. For proof of principle, we illustrate using 2,982 selected genes that include the “S1500+”^1^ gene-set. Induced pluripotent stem cells (iPSC)-cardiomyocytes were treated with three different doses of four chemicals to assess their effects on gene expression and concentration–response point of departure (POD) (GEO accession number GSE105050).

## Materials and Methods

### Chemicals and Biologicals

iCell cardiomyocytes (cat. no.: CMC-100-010-001), and cardiomyocyte plating and maintenance media were purchased from Cellular Dynamics International (Madison, WI, United States). Reference chemicals isoproterenol (CAS: 7683-59-2) and propranolol (CAS: 525-66-6) were purchased as part of EarlyTox Cardiotoxicity Screening kits (cat. no.: R8211) from Molecular Devices LLC (Sunnyvale, CA, United States). Nifedipine (CAS: 21829-25-4) and dofetilide (CAS: 115256-11-6) were purchased from APEXBio (Houston, TX, united States). Dimethyl sulfoxide (DMSO, cat. no.: sc-358801, CAS: 67-68-5) was purchased from Santa Cruz Biotechnology (Dallas, TX, United States). Penicillin/streptomycin solution (cat. no.: 10378016) and 0.4% Trypan Blue solution (cat. no.: 15250061) were obtained from Life Technologies (Grand Island, NY, United States).

### Cardiomyocyte Cell Culture

iCell cardiomyocytes were plated and maintained according to the manufacturer’s recommendations (Cellular Dynamics International, Madison, WI, United States) and in accordance with previously published protocols with minor adjustments (Sirenko et al., 2013a,b, 2017; Grimm et al., 2015, 2016). Individual units of cardiomyocytes were thawed for 4 min in a 37°C water bath and subsequently resuspended in 10 ml of cardiomyocyte plating medium containing 1:500 penicillin/streptomycin solution. Following microscopic cell counting using the trypan blue exclusion method, the cell density was adjusted to a final plating density of 2 × 10^5^ cells/ml. Twenty-five microliters of cell suspension was then transferred per well to a 384-well microplate, yielding a final cell density of 5,000/well. Tissue-culture treated microplates (cat. no.: 353962, Corning Life Sciences, Corning, NY, United States) were gelatinized for 2 h at 37°C with 25 μl 0.1% gelatin in water before cardiomyocytes were plated. After disposal of the gelatin solution and addition of the cell suspension, microplates were kept at room temperature for 30 min. Cells were then incubated at 37°C and 5% CO_2_ for 48 h. The plating medium was then exchanged with 40 μl of maintenance medium containing 1:500 penicillin/streptomycin solution per well. Maintenance medium was replaced every 48–72 h until day 13 post-plating. The maintenance medium was then exchanged with 50 μl fresh medium per well and incubated overnight. Cells were treated the next morning (day 14 post-plating) with 12.5 μl 5× chemical solutions in 0.5% DMSO (v/v) in media (vehicle) in addition to untreated or vehicle-treated negative control wells, and incubated at 37°C and 5% CO_2_. Following 24 h of incubation, the cell medium was discarded, and cardiomyocytes were lysed with 10 μl 1× lysis buffer provided in the TempO-Seq assay kit (BioSpyder Technologies, Inc., Carlsbad, CA, United States). Lysate-containing microplates were agitated at 300 rpm using a benchtop microplate shaker and stored at -80°C until further use.

### TempO-Seq Library Preparation and Sequencing

Differential gene expression patterns and concentration–response relationships were analyzed using TempO-Seq^TM^ (BioSpyder Technologies, Inc., Carlsbad, CA, United States) (Yeakley et al., 2017), a targeted RNA sequencing technology focused on a surrogate transcriptome panel comprising 2,982 transcripts, as described previously (Biopyder Toxpanel Library DO-01-096) (Grimm et al., 2016). The sequencing library was prepared according to the manufacturer’s guidelines and as previously described (Grimm et al., 2015). In brief, RNA in 2 μl of each cell lysate was hybridized with the provided detector oligo pool mix (2 μl per sample) using the following thermocycler settings: 10 min at 70°C, followed by gradual decrease to 45°C over 49 min, and ending with 45°C for 1 min. Subsequent steps included nuclease digestion (90 min at 37°C) ligation step (60 min at 37°C, followed by heat denaturation at 80°C for 30 min) following addition of 24 μl nuclease mix and 24 μl ligation mix. Ten microliters of each ligation product was then transferred to a 96-well amplification microplate containing 10 μl of PCR mix per well. The ligation products were then uniquely labeled during product amplification, when well-specific, “barcoded” primer pairs were introduced to templates. Sequence-based barcoding is an essential step allowing for correct identification and recognition of transcript-specific sequencing counts. Five microliters of sample amplicons from each well was subsequently pooled into a single sequencing library. The TempO-Seq library was further processed using a PCR clean-up kit (Clontech, Mountain View, CA, United States) prior to sequencing at Texas A&M University Genomics & Bioinformatics Services. Sequencing was achieved using a 50 single-end read mode in a rapid flow cell (two sequencing lanes for increased sequencing depth; mean reads per gene = 212) on a HiSeq 2500 Ultra-High-Throughput Sequencing System (Illumina, San Diego, CA, united States). The high-expression genes listed in Supplementary Table 1 were attenuated to allow for more sequencing depth. Sequence cluster identification, quality pre-filtering, base calling, and uncertainty assessment were conducted in real time using Illumina’s HCS 2.2.68 and RTA 1.18.66.3 software with default parameter settings. Sequencing readouts were demultiplexed to generate FASTQ files, and passed all internal quality controls (GEO accession number GSE105050).

### Temposeqcount Application: Availability and Implementation

*Temposeqcount* installs all dependencies in a Python virtual environment. It is released as an open-source software under the GNU General Public License and available from https://github.com/demis001/temposeqcount. Complete installation instructions are provided. Note that a unix operating system is required for this portion only.

### Pathway Analysis

The log_2_(fold change) (l2fc) and *p*-values from DESeq2 for dofetilide and nifedipine were analyzed through the use of IPA (Ingenuity^®^ Systems^2^). A core analysis was run using: User Dataset as reference, cutoff of *p* < 0.05, and all other values as default. The cardiac-related pathways (with *p*-value < 0.05) for *Tox Functions under Diseases* and *Functions* were chosen for Supplementary Figure 2.

### Differential Gene Expression and Concentration Response

Sample dataset, hash file, figures, and R Scripts for generating all figures and processes are available from https://github.com/jshousephd/HT-CBA.

## Results and Discussion

### Process Overview

The transcriptomic analysis methods discussed here focus on assessment of differentially expressed genes (DEGs) and concentration–response assessment using counts from TempO-Seq experiments. However, the methods and processes described herein are extensible to most types of count data. The sample dataset used and provided for illustration consists of 2 sets of 12 vehicle controls and 4 chemical treatments at three concentration levels (0.1, 1.0, and 10 μM). Two inotropic agents, the β-adrenergic receptor agonist and antagonist isoproterenol and propranolol, are known negative controls for cardiac QT prolongation. Nifedipine is a calcium channel blocker used to treat hypertension and dofetilide is an antiarrhythmic.

**FIGURE 1:**
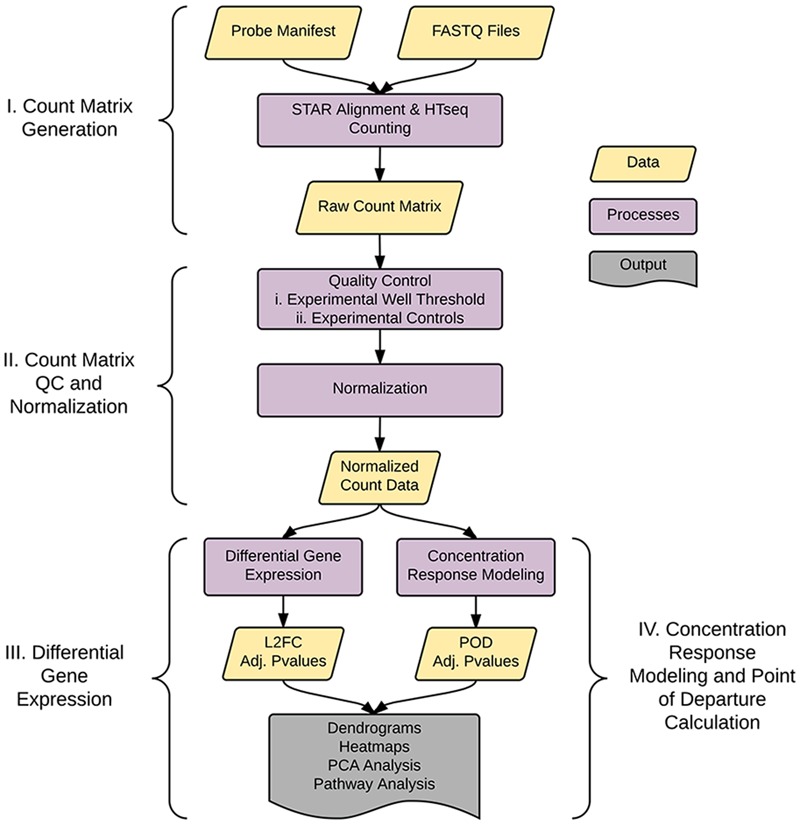
Pipeline overview. The pipeline consists of four major parts: I. Generation of the count matrix from sequenced reads, II. Quality-control and generation of normalized counts, III. Identification of differentially expressed genes (DEGs), and IV. Assessment of point of departure (POD) from count data.

As shown in **Figure 1**, the analysis pipeline can be broken into four major steps: (1) generation of the count matrix from sequenced reads (coded in *python*), (2) quality control and count normalization, (3) identification and visualization of DEGs, and (4) concentration–response modeling and POD assessment. POD assessment combines output from *tcpl* (Filer et al., 2017) augmented with additional model fitting as described below.

#### TempO-Seq Count Matrix Generation

Prior to assessment of DEGs and concentration response modeling, raw sequenced reads are aligned to probe sequences and counted. TempO-Seq is a high-throughput targeted sequencing technology that uses template-dependent oligo ligation on a multi-well plate. Generation of a count matrix from TempO-Seq data requires far less computing resources than traditional whole-transcriptome RNA-seq. Since the reads are generated from targeted probes, the reference file is several orders of magnitude smaller than a genome reference. For this application, sequencing reads were de-multiplexed by the sequencing facility. After de-multiplexing, a single experimental layout can result in up to 384 or 1,536 fastq files (depending on plate format), each file with reads resulting from an experimental condition. In traditional RNA-seq, individual fastq files are each aligned to a reference sequence individually using a short read aligner such as STAR, BWA, or bowtie (Langmead et al., 2009; Li and Durbin, 2010; Dobin et al., 2013) and then counted using a different command line utility. However, these routines are less useful in a TempO-Seq experiment due to the large number of fastq files generated in a single Tempo-Seq run and the provision of reference sequences.

**FIGURE 2:**
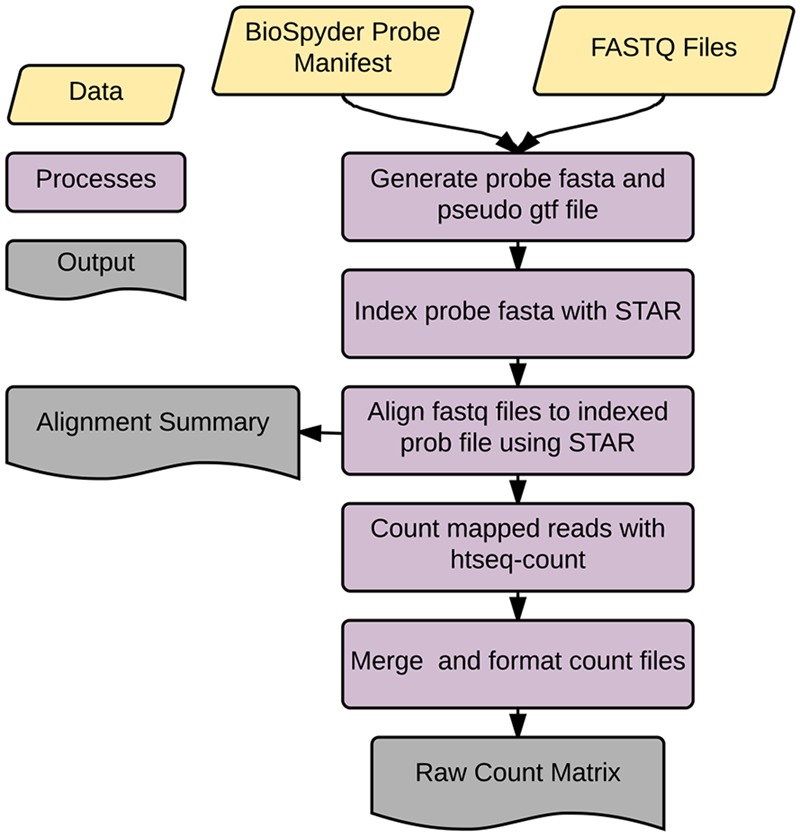
*temposeqcount* overview. Self-contained and implemented in a Ruffus framework. A directory of fastq files and the probe manifest are the only inputs to generate the count matrix required for the remainder of the pipeline.

Accordingly, we developed an application called *temposeqcount* to facilitate this process using a Ruffus framework in Python (Goodstadt, 2010) which is illustrated in **Figure 2**. Briefly, the application accepts the manufacturer provided probe manifest CSV and a directory of fastq files as input. Initially, the probe sequence is parsed from the manifest file which generates the probe fasta and pseudo-gtf annotation files. The probe fasta file is indexed using *genomeGenerate* function in the STAR aligner (Dobin et al., 2013) and STAR aligner is used to align a fastq file to indexed probe sequences. Lastly, htseq-count in the HTSeq (Anders et al., 2015) application is used to count probe-aligned reads. The *temposeqcount* application accepts STAR aligned bam and pseudo annotation-gtf files internally to generate a count for each sample. As output, an alignment summary is generated and count files are merged and formatted into a single count matrix (*K*) consisting of gene*_i_* rows and treatment*_j_* columns for downstream analysis.

### QC and Normalization of Counts

**FIGURE 3:**
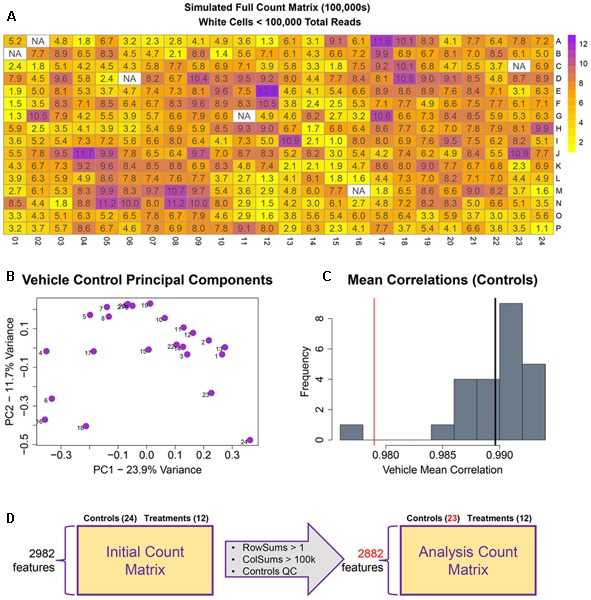
Raw count quality control assessment. **(A)** Simulated raw counts for a full 384-well TempO-Seq experiment. **(B)** First two principal components of vehicle controls. **(C)** Histogram of sample correlations for all vehicle controls. Black bar is the mean of sample correlations across all genes. Red bar represents 3 standard deviations from mean. **(D)** Summary of QC changes to the analysis matrix. 100 features were dropped due to <2 counts across all columns (2982–2882) and one control was dropped (leaving 23) due to low correlation with other control samples. No samples had <100 k counts.

The remainder of the pipeline (**Figure 1**; steps II, III, and IV) consists of R scripts that are publicly available^3^. The inputs to the rest of the pipeline consist of the count matrix (*K*) generated in step I, and an experimental layout file (hereafter referred to as hash file) from the experimenter. Although we are using this process for counts from the TempO-Seq assay, the pipeline here can be applied to other types of high-throughput sequencing data. Multiple attributes can be included in the hash file for each treatment, but column names of the count matrix must have a corresponding column entry in the hash file. Gene features are filtered for >1 count per row across the experimental count matrix, which resulted in 100 genes removed, leaving 2,882 for subsequent analyses (**Figure 3D**). Prior to normalization, sample count totals are evaluated graphically (**Figure 3A**) and samples (columns) failing to exceed a user defined minimum count threshold (we used 100,000 per sample for the library of 2,982 features in the TempO-seq kits used in these experiments) are removed from subsequent analyses. The design of concentration–response experiments for multiple compounds often uses shared controls. For the data examined herein, there were 24 vehicle controls. In the pipeline, controls are examined by principal component analysis (PCA; **Figure 3B**). In addition, we examine the average correlations of each control sample with the remaining samples (**Figure 3C**), an approach termed the “D statistic” and similar to that used by the GTEx Consortium (Consortium, 2015) to filter low-quality samples. In this analysis, we removed control samples for which the average correlation with remaining control samples was 3 standard deviations lower than a mean computed for all controls (**Figure 3C**, red line). Pairwise correlations and scatterplots were also examined for controls prior to normalization (Supplementary Figure 1). The final analysis count matrix (**Figure 3D**) was then normalized experiment-wide at the treatment level with DESeq2, which models read counts using a negative binomial distribution and normalizes based on a model that uses dispersion estimates for each gene across all treatments (Love et al., 2014).

### Analysis of Differential Gene Expression

**FIGURE 4:**
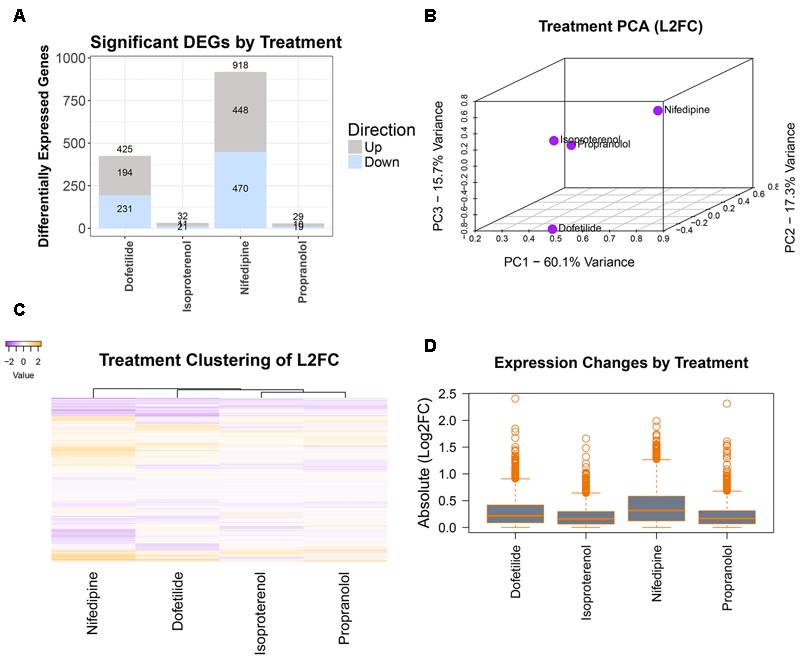
Differential gene expression assessment. Cleaned count data are normalized and assessed for DEGs (at max treatment dose) by treatment using DESeq2. Magnitude of transcriptomic effects and comparison of chemicals are illustrated in: **(A)** number of DEGs in each treatment, **(B)** PCA of the log_2_(fold changes), **(C)** heatmap of the log_2_(fold changes), and **(D)** mean magnitudes and dispersions of the absolute log_2_(fold changes).

Differentially expressed genes were determined using DESeq2 prior to concentration response modeling. DEGs were first identified for the maximum concentration of each treatment, with log_2_ fold change (l2fc) values, p-values, and adjusted p-values computed for each gene and combined into a single dataset for each chemical. For the sample data, as seen in the summary plot of the number of DEGs per treatment (**Figure 4A**), nifedipine was the most transcriptionally active treatment with identified differential gene expression for nearly a third of interrogated transcripts (918/2,882). Dofetilide affected 425 transcripts, while isoproterenol and propranolol had little effect on transcription, with 32 and 29 identified DEGs, respectively (**Figure 4A**).

A typical TempO-Seq experiment may have 50–75 unique concentration-chemical combinations making it important to examine how they group together regarding differential gene expression. There are several ways to do this, and we have illustrated some of them in **Figure 4** with the sample dataset. Using all l2fc values for each chemical, the first three principal components were plotted (**Figure 4B**). In the sample data, the two drugs with effects on the heart rhythm (isoproterenol and propranolol) clustered together while the others (nifedipine and dofetilide) were quite distinct. A heatmap of l2fc values further illustrates the similarities between the isoproterenol and propranolol, while highlighting how both nifedipine and dofetilide are each different from these QT-prolongation controls and from each other (**Figure 4C**). The overall magnitude of transcription effects from each chemical is shown in the boxplot of absolute l2fc values (**Figure 4D**).

### Concentration–Response Modeling Decision Logic

**FIGURE 5:**
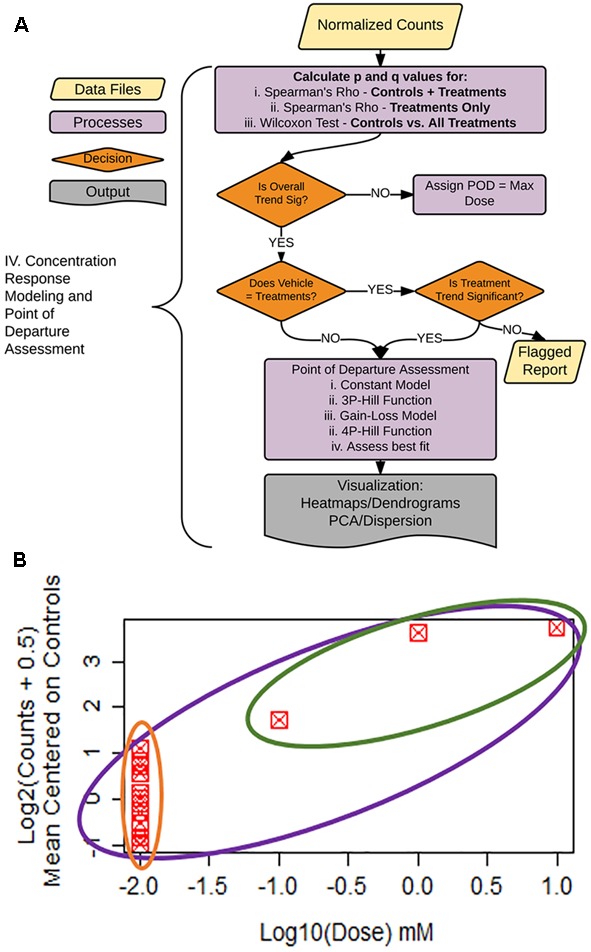
Statistical flag generation for concentration-response modeling. **(A)** Flowchart of decision logic used in determining the gene-treatment combinations to assess for concentration response. **(B)** Example concentration–response curve used for statistical flag generation. Concentration–response relationships where overall ρ (purple circle) is significant, and controls not equal to treatments (green vs. orange), are chosen for subsequent concentration response modeling, as are relationships where ρ for treatments only (green circle) are significant.

A critical step in human health assessment for a chemical compound is the determination of the POD from a dose/concentration–response relationship. In the sample data, iPSC cardiomyocytes were exposed to either vehicle or three increasing drug concentrations (0.1, 1.0, and 10 μM). Vehicle controls were assigned a dose value on the log_10_ scale that is one average dose distance below the lowest treatment concentration (**Figure 5B**). Our dataset contained 24 vehicle controls and a single treatment at each of the three concentrations. Thus, since one control was removed in QC, the dosing vector for this experiment consists of 23 values of -2, followed by -1, 0, and 1, while the response vector consists of normalized counts data at each control/treatment. To allow for zero counts, normalized counts were log_2_(counts + 0.5) transformed and mean-centered to vehicle controls. An example plot of the data is shown in **Figure 5B**.

To facilitate decision-making accompanying our concentration–response modeling, we created a process tree that utilizes statistical flags (**Figures 5A,B**). The q-values (FDR values for each p-value threshold), are calculated for: (1) the moment-corrected correlation (MCC) trend test (Zhou and Wright, 2015) for the entire concentration–response range (**Figure 5B**, purple oval), (2) MCC for treatment doses only (**Figure 5B**, green oval), and (3) Wilcoxon’s statistic for a difference between vehicle control values vs. treatments (**Figure 5B** green oval vs. orange oval). MCC is a trend procedure that is intended to be powerful while retaining robustness to a wide variety of distributional forms of the data test (Zhou and Wright, 2015). These flags were used to decide which gene/treatment combinations should be fit for a concentration–response (**Figure 5A**). If the overall trend (**Figure 5B**, purple) in the concentration–response is not significant, the experimental maximum dose is assigned as the POD. For those genes where the overall trend is significant, control counts are compared to treatment counts using a Wilcoxon Rank Sum test. If these two groups are different from each other (**Figure 5B**, green vs. orange), the gene/treatment is selected for concentration–response modeling. If controls are not different from treatments but did pass the overall trend test for significance, we then assess whether a trend exists in treatments only (**Figure 5B**, green). If the treatment-only trend is significant, it is also selected for POD modeling. For those remaining genes that are not significant for the treatment only trend test, a report is generated for the remaining items for manual experimenter follow-up.

### Concentration–Response Modeling and Point of Departure Calculation

**FIGURE 6:**
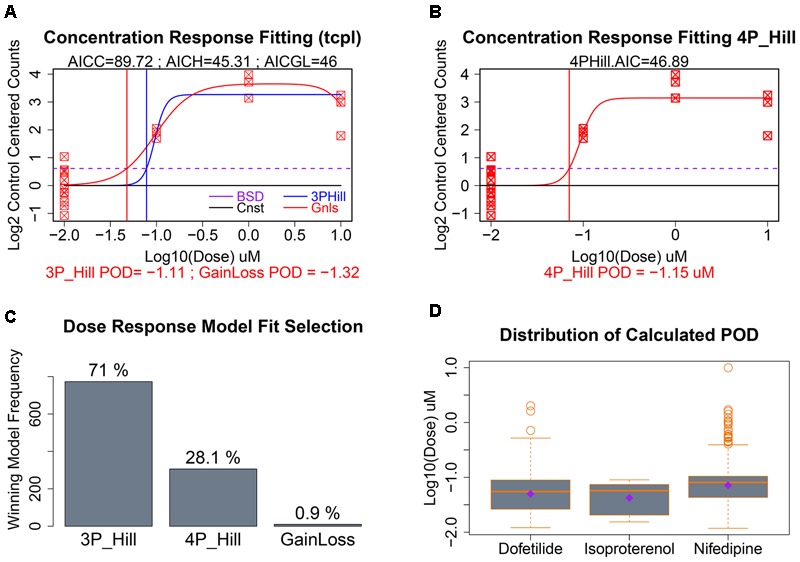
Concentration–response modeling and POD calculation. **(A)** Baseline deviation (BSD) is set to 1 SD from mean of controls (dotted purple line). The tcpl package is used to fit (1) constant, (2) gain loss, and (3) three-parameter hill models. **(B)** The DRC package in R is used to fit a four-parameter hill function and the model with the smallest AIC is chosen as “best” with corresponding POD used where fitted model crosses BSD. **(C)** Distribution of the winning model for these data. **(D)** Evaluation of the dispersion, mean, and median of calculated POD deviations by chemical treatment.

As seen in **Figure 5B**, our data had only a single replicate at each of three concentrations of a given chemical. To illustrate clearly dose response modeling we show simulated data for 12 control replicates and three concentrations with three replicates each (**Figures 6A,B**). For those gene/treatments identified for concentration–response modeling, “dose” vectors and response counts are first fit with *tcpl* functions (Filer et al., 2017) with a constant model that represents a null fit, a gain-loss model, and a three-parameter hill model with the “floor” set to zero (**Figure 6A**). We also assess a four-parameter hill function fit using the R-DRM package (Ritz et al., 2015) where the “floor” is not set to zero, and assess the best-fitting model by the smallest Akaike information criterion (AIC) which penalizes model over-fitting. Following selection of the best model, the POD is assessed by determining the concentration that elicits a 1 standard deviation departure from the control mean (**Figures 6A,B**; dotted purple line), although other POD approaches or benchmark dose (Sirenko et al., 2013b; Wignall et al., 2014; Filer et al., 2017) could be used. For the sample with only three treatment concentrations, the three-parameter hill function was the predominant “winner” in terms of minimum AIC, although a four-parameter hill function provides the best fit in 28% of the fitted curves (**Figure 6C**). Although nifedipine caused more than twice the number of differentially expressed genes as dofetilide (**Figure 4A**), dofetilide actually had a smaller mean POD (**Figure 6D**). Propranolol is not shown in **Figure 6D** as it had no genes exhibiting concentration–response relationships.

TempO-seq is a new technology with few publications to date and no standard pipeline for analysis. A recent study by Yeakley et al. (2017) (GEO GSE91395_Dose_Response_Read_Counts.xlsx) also followed a concentration–response study design. We thus used their data as another highlight speed and utility of our pipeline. First, using our pipeline we found 4,178 genes that were differentially expressed in MCF-7 cells treated with 1 μM Trichostatin A, while Yeakley et al. (2017) had reported 4,154 differentially expressed genes. We then used our concentration–response pipeline to calculate POD estimates for several top upregulated genes and two of the novel genes they reported for Trichostatin A response. These are graphically represented in Supplementary Figure 2.

## Conclusion and Summary

As sequencing has become more affordable, the number of experiments with sequencing (expression) data has grown exponentially, and multiple computational tools are available for different steps in the analysis. Similarly, many dose–response modeling tools are available (Wignall et al., 2014; Filer et al., 2017; Sirenko et al., 2017), including for gene expression data at the level of genes and pathways (Yang et al., 2007).

Gene expression data have been an important contributor to the mechanistic studies in toxicology and other biomolecular fields (Luo et al., 2017). Toxicogenomics is a valuable tool for predictive modeling (Uehara et al., 2008; De Abrew et al., 2015) and read-across (Low et al., 2011; Grimm et al., 2016). However, the high cost of gene expression studies has largely precluded the use of toxicogenomics as a standard tool for dose–response assessment in studies of adverse effects of drugs and chemicals. While some databases do include dose- and time-dependent profiling of hundreds of drugs and chemicals (Luo et al., 2017), the potential power of dose–response expression profiling has not been fully harnessed. The potential of dose–response toxicogenomics data as a truly predictive tool was first demonstrated by Thomas et al. (2011, 2013), who showed that gene expression data-based PODs derived from short-term studies are well-correlated with the PODs for the apical endpoints from 90-day and 2-year animal studies (Farmahin et al., 2017). A recent study demonstrated the value of dose–response genomics in a comparative analysis of chlorinated solvents in liver and kidney (Zhou et al., 2017). Thus, with the advent of lower cost and higher throughput platforms for gene expression profiling, dose–response modeling will become a major output of these experiments, including in *in vitro* studies.

The methods outlined in this manuscript provide a framework for highly automated assessment of transcriptomic concentration–response POD estimates. Although we have used targeted sequencing data, these methods are extensible to any kind of concentration–response count data, including whole-transcriptome RNA sequencing. Probe-based targeted RNA-seq technology does have many advantages. These procedures are extremely fast and can be run on desktop computers instead of computing clusters. The biases associated with the purification and library creation in RNA-seq are not applicable with this technology. However, it is important to note that this targeted RNA-seq technology does not capture underlying genetic variation in a region of interest. The probes are highly specific to the 50-mer being interrogated. The methods described here have been made freely available, to provide tools to characterize the transcriptomic dose response and concentration response effects of drugs and chemicals in novel targeted-probe high-throughput formats.

The gene expression response signatures identified by our pipeline can be used for hazard assessment, drug repurposing, and disease characterization (Stegmaier et al., 2004; Hieronymus et al., 2006; Wei et al., 2006; Sirota et al., 2011). We note that agonism is the characterized mode of action for isoproterenol and propranolol, and antagonism of the β-adrenergic receptor for positive and negative inotropic effects. We report few transcriptional effects from treatment with either of these drugs. One possible interpretation is the presence of very little non-receptor-mediated mode of action. In contrast, treatment of iPSC cardiomyocytes with nifedipine, a calcium channel blocker used to treat hypertension, resulted in substantial transcriptional changes. Dofetilide, an antiarrhythmic agent that increases the QT interval by selectively blocking the rapid component of the cardiac ion channel delayed rectifier current (Roukoz and Saliba, 2007), also resulted in substantial transcriptional changes. Further extension of these results into pathway analysis and biological read across will facilitate additional decision-making processes for hazard and risk characterization, drug repurposing, and hypothesis formation. Although our sample data provided contained few replicates at each dose, we still were able to identify DEGs and calculate POD estimates. As a proof of principle, we examined DEGs for dofetilide and nifedipine using Ingenuity’s Pathway Analysis. The top identified toxicology pathways were heart and liver related for dofetilide, and more diverse for nifedipine. The statistically significant overlapping cardiac-related pathways are shown in Supplementary Figure 3.

In summary, with the advent of cheaper sequencing technology and targeted sequencing technology, it has become feasible and necessary to utilize the value of gene expression data in high-throughput experiments, for dose response characterization, for perturbation signature identification, and for biological read-across assessment. The methods herein provide a reproducible, largely automated framework to utilize such sequencing data to identify treatment-induced DEGs and concentration response estimates.

## Author Contributions

JH: experimental design, methodology development, programming, writing and editing, and submitting. FG: experimental design, all wet-lab work, and writing and editing. DJ: experimental design, methodology development, programming, and writing and editing. Y-HZ: methodology development. IR: study conception, experimental design, support, and writing and editing. FW: study conception, experimental design, support, writing and editing.

## Conflict of Interest Statement

The authors declare that the research was conducted in the absence of any commercial or financial relationships that could be construed as a potential conflict of interest.

## Footnotes

1 https://federalregister.gov/a/2015-08529

2 www.ingenuity.com

3 https://github.com/jshousephd/HT-CBA

## References

[B1] Anders S. Pyl P. T. Huber W. (2015). HTSeq–a python framework to work with high-throughput sequencing data. *Bioinformatics* 31 166–169. 10.1093/bioinformatics/btu638 25260700 PMC4287950

[B2] Campillos M. Kuhn M. Gavin A. C. Jensen L. J. Bork P. (2008). Drug target identification using side-effect similarity. *Science* 321 263–266. 10.1126/science.1158140 18621671

[B3] Consortium G. T. (2015). Human genomics. The genotype-tissue expression (GTEx) pilot analysis: multitissue gene regulation in humans. *Science* 348 648–660. 10.1126/science.1262110 25954001 PMC4547484

[B4] De Abrew K. N. Overmann G. J. Adams R. L. Tiesman J. P. Dunavent J. Shan Y. K. (2015). A novel transcriptomics based in vitro method to compare and predict hepatotoxicity based on mode of action. *Toxicology* 328 29–39. 10.1016/j.tox.2014.11.008 25475144

[B5] Dobin A. Davis C. A. Schlesinger F. Drenkow J. Zaleski C. Jha S. (2013). STAR: ultrafast universal RNA-seq aligner. *Bioinformatics* 29 15–21. 10.1093/bioinformatics/bts635 23104886 PMC3530905

[B6] Duan Q. Flynn C. Niepel M. Hafner M. Muhlich J. L. Fernandez N. F. (2014). LINCS canvas browser: interactive web app to query, browse and interrogate LINCS L1000 gene expression signatures. *Nucleic Acids Res.* 42 W449–W460. 10.1093/nar/gku476 24906883 PMC4086130

[B7] Ellis S. E. Gupta S. Ashar F. N. Bader J. S. West A. B. Arking D. E. (2013). RNA-Seq optimization with eQTL gold standards. *BMC Genomics* 14:892. 10.1186/1471-2164-14-892 24341889 PMC3890578

[B8] Farmahin R. Williams A. Kuo B. Chepelev N. L. Thomas R. S. Barton-Maclaren T. S. (2017). Recommended approaches in the application of toxicogenomics to derive points of departure for chemical risk assessment. *Arch. Toxicol.* 91 2045–2065. 10.1007/s00204-016-1886-5 27928627 PMC5399047

[B9] Filer D. L. Kothiya P. Setzer R. W. Judson R. S. Martin M. T. (2017). tcpl: the ToxCast pipeline for high-throughput screening data. *Bioinformatics* 33 618–620. 10.1093/bioinformatics/btw680 27797781

[B10] Goodstadt L. (2010). Ruffus: a lightweight Python library for computational pipelines. *Bioinformatics* 26 2778–2779. 10.1093/bioinformatics/btq524 20847218

[B11] Grimm F. A. Iwata Y. Sirenko O. Bittner M. Rusyn I. (2015). High-content assay multiplexing for toxicity screening in induced pluripotent stem cell-derived cardiomyocytes and hepatocytes. *Assay Drug Dev. Technol.* 13 529–546. 10.1089/adt.2015.659 26539751 PMC4652224

[B12] Grimm F. A. Iwata Y. Sirenko O. Chappell G. A. Wright F. A. Reif D. M. (2016). A chemical-biological similarity-based grouping of complex substances as a prototype approach for evaluating chemical alternatives. *Green Chem.* 18 4407–4419. 10.1039/c6gc01147k 28035192 PMC5179981

[B13] Han Y. Gao S. Muegge K. Zhang W. Zhou B. (2015). Advanced applications of RNA sequencing and challenges. *Bioinform. Biol. Insights* 9(Suppl. 1) 29–46. 10.4137/BBI.S28991 26609224 PMC4648566

[B14] Hieronymus H. Lamb J. Ross K. N. Peng X. P. Clement C. Rodina A. (2006). Gene expression signature-based chemical genomic prediction identifies a novel class of HSP90 pathway modulators. *Cancer Cell* 10 321–330. 10.1016/j.ccr.2006.09.005 17010675

[B15] Igarashi Y. Nakatsu N. Yamashita T. Ono A. Ohno Y. Urushidani T. (2015). Open TG-GATEs: a large-scale toxicogenomics database. *Nucleic Acids Res.* 43 D921–D927. 10.1093/nar/gku955 25313160 PMC4384023

[B16] Klebanov L. Chen L. Yakovlev A. (2007). Revisiting adverse effects of cross-hybridization in Affymetrix gene expression data: do they matter for correlation analysis? *Biol. Direct* 2:28. 10.1186/1745-6150-2-28 17988401 PMC2211459

[B17] Lamb J. Crawford E. D. Peck D. Modell J. W. Blat I. C. Wrobel M. J. (2006). The connectivity map: using gene-expression signatures to connect small molecules, genes, and disease. *Science* 313 1929–1935. 10.1126/science.1132939 17008526

[B18] Langmead B. Trapnell C. Pop M. Salzberg S. L. (2009). Ultrafast and memory-efficient alignment of short DNA sequences to the human genome. *Genome Biol.* 10:R25. 10.1186/gb-2009-10-3-r25 19261174 PMC2690996

[B19] Li H. Durbin R. (2010). Fast and accurate long-read alignment with Burrows-Wheeler transform. *Bioinformatics* 26 589–595. 10.1093/bioinformatics/btp698 20080505 PMC2828108

[B20] Li H. Qiu J. Fu X. D. (2012). RASL-seq for massively parallel and quantitative analysis of gene expression. *Curr. Protoc. Mol. Biol.* Chapter 4:Unit 4.13. 1–9. 10.1002/0471142727.mb0413s98 22470064 PMC3325489

[B21] Love M. I. Huber W. Anders S. (2014). Moderated estimation of fold change and dispersion for RNA-seq data with DESeq2. *Genome Biol.* 15:550. 10.1186/s13059-014-0550-8 25516281 PMC4302049

[B22] Low Y. Uehara T. Minowa Y. Yamada H. Ohno Y. Urushidani T. (2011). Predicting drug-induced hepatotoxicity using QSAR and toxicogenomics approaches. *Chem. Res. Toxicol.* 24 1251–1262. 10.1021/tx200148a 21699217 PMC4281093

[B23] Luo G. Shen Y. Yang L. Lu A. Xiang Z. (2017). A review of drug-induced liver injury databases. *Arch. Toxicol.* 10.1007/s00204-017-2024-8 [Epub ahead of print]. 28717830

[B24] Merrick B. A. Paules R. S. Tice R. R. (2015). Intersection of toxicogenomics and high throughput screening in the Tox21 program: an NIEHS perspective. *Int. J. Biotechnol.* 14 7–27. 10.1504/IJBT.2015.074797 27122658 PMC4844067

[B25] National Academies (2017). *Using 21st Century Science to Improve Risk-Related Evaluations.* Washington, DC: The National Academies Press. 28267305

[B26] National Research Council (2014). *A Framework to Guide Selection of Chemical Alternatives.* Washington, DC: The National Academies Press. 25473704

[B27] National Research Council (2007). *Toxicity Testing in the 21st Century.* Washington, DC: The National Academies Press.

[B28] Okoniewski M. J. Miller C. J. (2006). Hybridization interactions between probesets in short oligo microarrays lead to spurious correlations. *BMC Bioinformatics* 7:276. 10.1186/1471-2105-7-276 16749918 PMC1513401

[B29] Peck D. Crawford E. D. Ross K. N. Stegmaier K. Golub T. R. Lamb J. (2006). A method for high-throughput gene expression signature analysis. *Genome Biol.* 7:R61. 10.1186/gb-2006-7-7-r61 16859521 PMC1779561

[B30] Ritz C. Baty F. Streibig J. C. Gerhard D. (2015). Dose-response analysis using R. *PLOS ONE* 10:e0146021. 10.1371/journal.pone.0146021 26717316 PMC4696819

[B31] Roukoz H. Saliba W. (2007). Dofetilide: a new class III antiarrhythmic agent. *Exp. Rev. Cardiovasc. Ther.* 5 9–19. 10.1586/14779072.5.1.9 17187453

[B32] Royce T. E. Rozowsky J. S. Gerstein M. B. (2007). Toward a universal microarray: prediction of gene expression through nearest-neighbor probe sequence identification. *Nucleic Acids Res.* 35:e99. 10.1093/nar/gkm549 17686789 PMC1976448

[B33] Sirenko O. Crittenden C. Callamaras N. Hesley J. Chen Y. W. Funes C. (2013a). Multiparameter in vitro assessment of compound effects on cardiomyocyte physiology using iPSC cells. *J. Biomol. Screen.* 18 39–53. 10.1177/1087057112457590 22972846

[B34] Sirenko O. Cromwell E. F. Crittenden C. Wignall J. A. Wright F. A. Rusyn I. (2013b). Assessment of beating parameters in human induced pluripotent stem cells enables quantitative in vitro screening for cardiotoxicity. *Toxicol. Appl. Pharmacol.* 273 500–507. 10.1016/j.taap.2013.09.017 24095675 PMC3900303

[B35] Sirenko O. Grimm F. A. Ryan K. R. Iwata Y. Chiu W. A. Parham F. (2017). In vitro cardiotoxicity assessment of environmental chemicals using an organotypic human induced pluripotent stem cell-derived model. *Toxicol. Appl. Pharmacol.* 322 60–74. 10.1016/j.taap.2017.02.020 28259702 PMC5734940

[B36] Sirota M. Dudley J. T. Kim J. Chiang A. P. Morgan A. A. Sweet-Cordero A. (2011). Discovery and preclinical validation of drug indications using compendia of public gene expression data. *Sci. Transl. Med.* 3:96ra77. 10.1126/scitranslmed.3001318 21849665 PMC3502016

[B37] Stegmaier K. Ross K. N. Colavito S. A. O’Malley S. Stockwell B. R. Golub T. R. (2004). Gene expression-based high-throughput screening(GE-HTS) and application to leukemia differentiation. *Nat. Genet.* 36 257–263. 10.1038/ng1305 14770183

[B38] Thomas R. S. Clewell H. J. Allen B. C. Wesselkamper S. C. Wang N. C. Lambert J. C. (2011). Application of transcriptional benchmark dose values in quantitative cancer and noncancer risk assessment. *Toxicol. Sci.* 120 194–205. 10.1093/toxsci/kfq355 21097997

[B39] Thomas R. S. Wesselkamper S. C. Wang N. C. Zhao Q. J. Petersen D. D. Lambert J. C. (2013). Temporal concordance between apical and transcriptional points of departure for chemical risk assessment. *Toxicol. Sci.* 134 180–194. 10.1093/toxsci/kft094 23596260

[B40] Uehara T. Hirode M. Ono A. Kiyosawa N. Omura K. Shimizu T. (2008). A toxicogenomics approach for early assessment of potential non-genotoxic hepatocarcinogenicity of chemicals in rats. *Toxicology* 250 15–26. 10.1016/j.tox.2008.05.013 18619722

[B41] Uehara T. Ono A. Maruyama T. Kato I. Yamada H. Ohno Y. (2010). The Japanese toxicogenomics project: application of toxicogenomics. *Mol. Nutr. Food Res.* 54 218–227. 10.1002/mnfr.200900169 20041446

[B42] Wei G. Twomey D. Lamb J. Schlis K. Agarwal J. Stam R. W. (2006). Gene expression-based chemical genomics identifies rapamycin as a modulator of MCL1 and glucocorticoid resistance. *Cancer Cell* 10 331–342. 10.1016/j.ccr.2006.09.006 17010674

[B43] Wignall J. A. Shapiro A. J. Wright F. A. Woodruff T. J. Chiu W. A. Guyton K. Z. (2014). Standardizing benchmark dose calculations to improve science-based decisions in human health assessments. *Environ. Health Perspect.* 122 499–505. 10.1289/ehp.1307539 24569956 PMC4014768

[B44] Yang L. Allen B. C. Thomas R. S. (2007). BMDExpress: a software tool for the benchmark dose analyses of genomic data. *BMC Genomics* 8:387. 10.1186/1471-2164-8-387 17961223 PMC2198920

[B45] Yeakley J. M. Shepard P. J. Goyena D. E. VanSteenhouse H. C. McComb J. D. Seligmann B. E. (2017). A trichostatin A expression signature identified by TempO-Seq targeted whole transcriptome profiling. *PLOS ONE* 12:e0178302. 10.1371/journal.pone.0178302 28542535 PMC5444820

[B46] Zhou Y. H. Cichocki J. A. Soldatow V. Y. Scholl E. Gallins P. Jima D. (2017). Comparative dose-response analysis of liver and kidney transcriptomic effects of trichloroethylene and tetrachloroethylene in B6C3F1 mouse. *Toxicol. Sci.* 10.1093/toxsci/kfx165 [Epub ahead of print]. 28973375 PMC5837274

[B47] Zhou Y. H. Wright F. A. (2015). Hypothesis testing at the extremes: fast and robust association for high-throughput data. *Biostatistics* 16 611–625. 10.1093/biostatistics/kxv007 25792622 PMC4804120

