# Supplementary material for: A Pipeline for High-Throughput Concentration Response Modeling of Gene Expression for Toxicogenomics
Source: Front Genet. 2017 Nov 1;8:168. doi: 10.3389/fgene.2017.00168 (PMC5672545; doi:10.3389/fgene.2017.00168)
Supplement: Supplementary file 1 [file Image_1.PDF]

Supplementary Material

A Pipeline for High Throughput Concentration Response Modeling of Gene Expression for Toxicogenomics

John S. House, Fabian A. Grimm, Dereje D. Jima, Yi-Hui Zhou, Ivan Rusyn, Fred A. Wright

Supplemental Table 1. TempOseq Attenuators

Attenuated Genes				
HP	ATP1B1	RPLP0	SKP1	ATP5B
ALB	RPS18	HBG2	RPL28	HIST1H2BD
FGG	RPS3	UBB	HIST1H1E	GAPDH
FGB	HSPB1	VIM	ND6	ORM1
SPP1	SAA1	HIST1H2BC	RPS7	HIST1H3H
RPL32	RPLP0	RPL15	HIST2H2AA4	

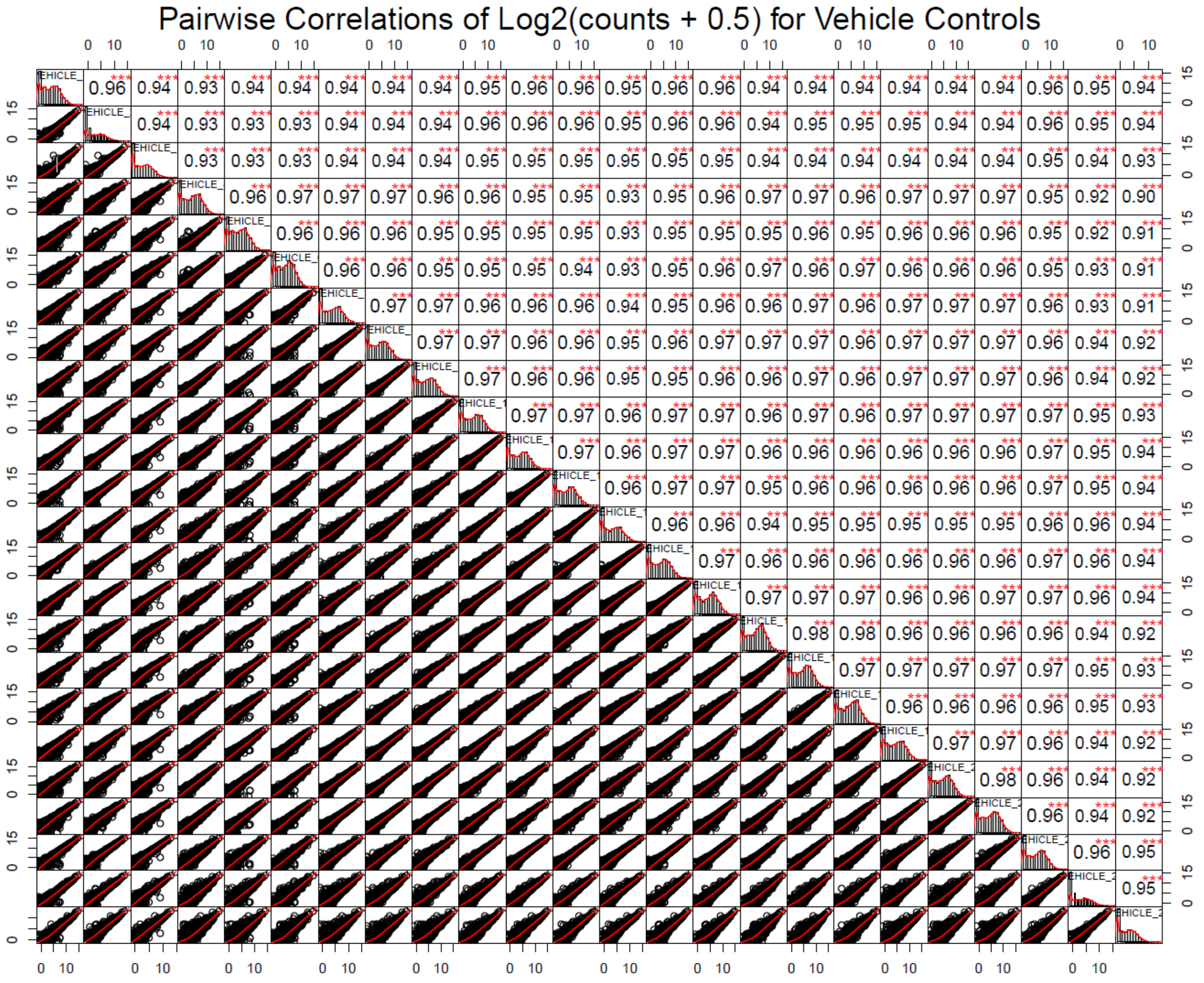

Supplementary Figure 1: Control Correlations. Vehicle controls were zero-protected and $\log_2$ transformed before examining pairwise Spearman correlations. Figure represents the 24 controls for the TempO-Seq experiment.

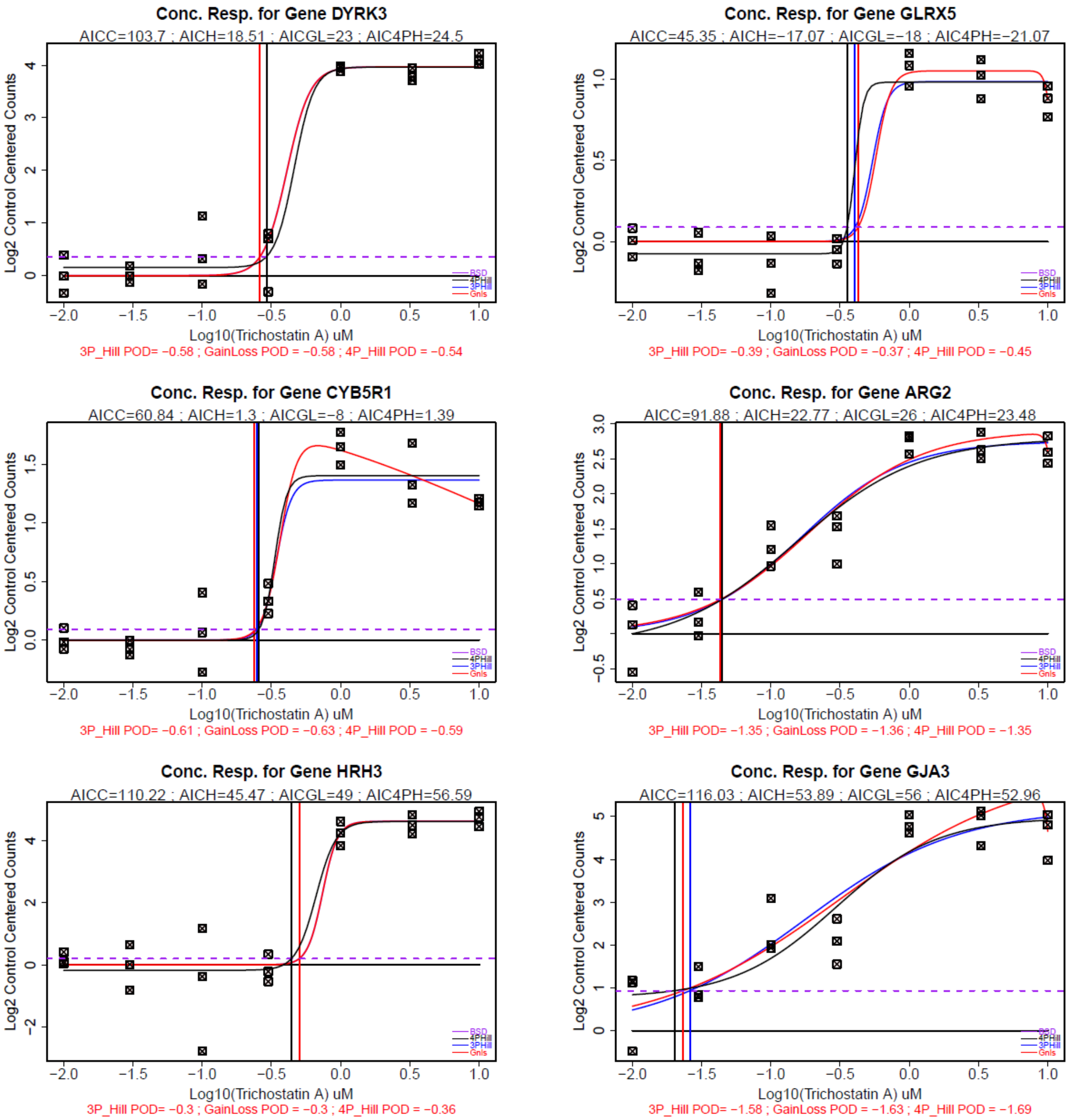

Supplementary Figure 2: Concentration Response Modeling using Yeakley et.al data. Modeling of gain-loss, 3-parameter Hill, 4-parameter Hill, and constant models for the top 4 trichostatin-A (TSA) up-regulated genes (log10(dose µM) = 0 for DYRK3, GLRX5, CYB5R1, and ARG2)) and for two of their newly reported responsive genes (HRH3 and GJA3). Calculated POD would be the one with the lowest AIC.

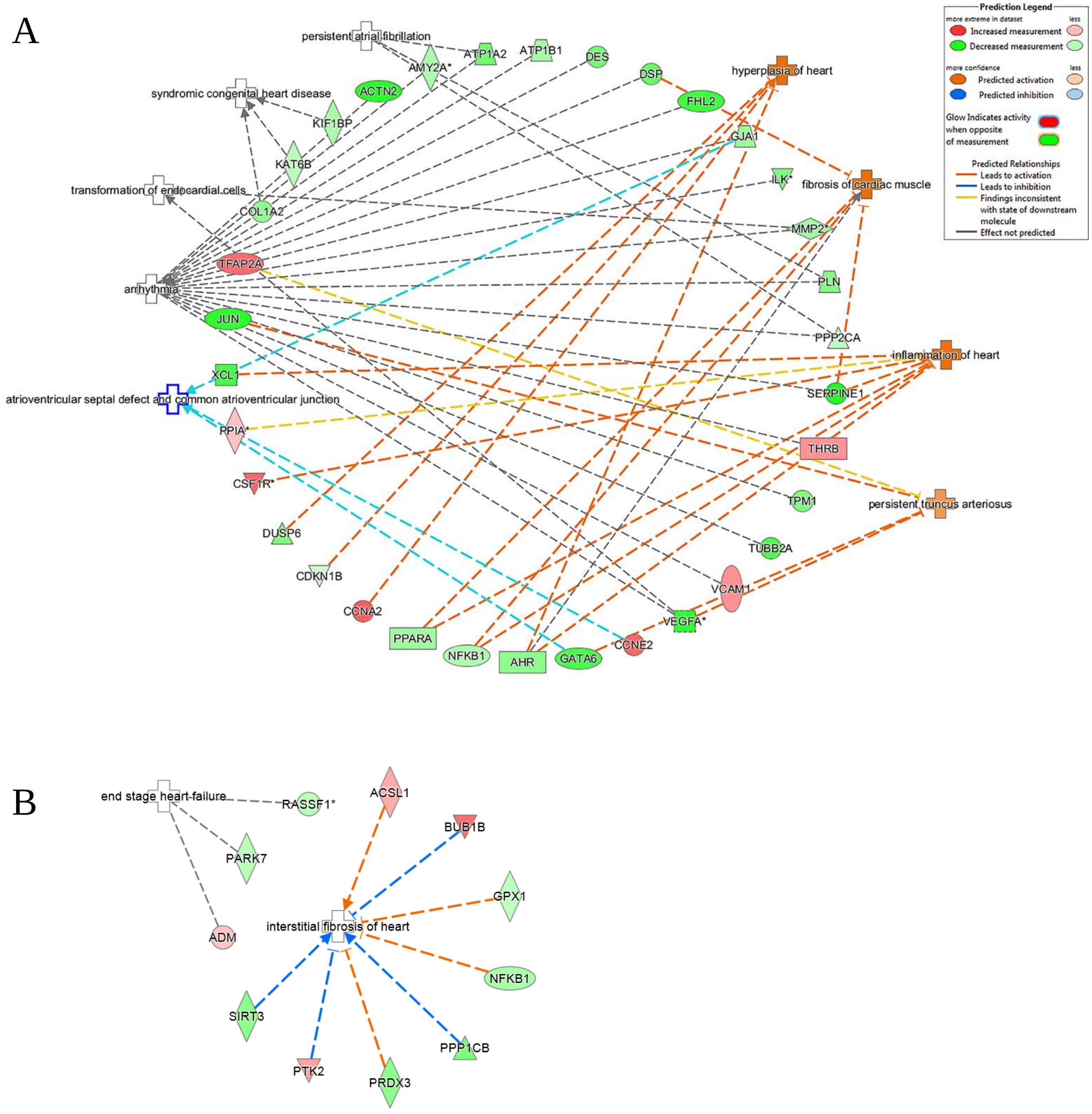

Supplementary Figure 3: Cardiac Related Pathways. Log_2(fold change) values and p-values for dofetilide (A) and nifedipine (B) were uploaded to Ingenuity Pathways Analysis. Predicted activation of inflammatory, fibrotic and hyperplasia of heart as well as persistent truncus arteriosus are shown in orange. Additional cardiac related effects in pathways related to heart development and function are shown in white.
